# Sudden Death After Endoscopic Retrograde Cholangiopancreatography (ERCP)—Case Report and Literature Review

**DOI:** 10.1097/MD.0000000000000235

**Published:** 2014-12-12

**Authors:** Goran Hauser, Marko Milosevic, Marko Zelić, Davor Stimac

**Affiliations:** From the Department of Internal Medicine, Division of Gastroenterology (GH, DS); Department of Surgery, Division of Digestive Surgery (MZ); and Department of Anaesthesiology, Clinical Hospital Centre Rijeka, 51000 Rijeka, Croatia (MM).

## Abstract

There are only a few cases found in literature regarding air embolism in endoscopic procedures, especially in connection to endoscopic retrograde cholangiopancreatography (ERCP). We are presenting a case of a 56-year-old female patient who suffered from non-Hodgkin lymphoma located in her right groin. She was also diagnosed with choledocholithiasis and underwent ERCP to remove the gallstones. Immediately after the procedure she went into sudden cardiac arrest and subsequently died, despite all of our efforts. We reviewed literature in order to identify possible causes of death because fatal outcome following an uneventful and successful procedure was not expected. It is important to bear in mind all possible complications of ERCP. Our focus during the literature search was on air embolism.

## INTRODUCTION

Endoscopic retrograde cholangiopancreatography (ERCP) is a widely used procedure to both diagnose and treat diseases of the pancreatobiliary tree, with over 500,000 ERCP procedures performed annually in the USA alone.^1^ Although this procedure is complex and requires great skill, it is performed routinely on a daily basis. Because of its complexity it carries some risks of complications. Most common complications of endoscopic retrograde cholangiopancreatography are hemorrhage, pancreatitis, cholangitis and perforation.^2^ Other, less common, complications include cardiac arrest, air in hepatic veins, cerebral air embolism and paradoxical air embolism.^3^ We are presenting a case of a 56-year-old female with sudden cardiac arrest and subsequent death of unknown etiology. Specificity of this case is in the fact that a patient underwent a routine endoscopic procedure and died. Especially if we take into consideration it was during the procedure, but after it had finished uneventfully. Due to many controversies in this field, we reviewed recent literature in order to detect possible causes of preventable death.

## CASE REPORT

A 56-year-old female patient with localized non-Hodgkin lymphoma was referred to a gastroenterologist prior to scheduled chemotherapy due to cholestatic profile in laboratory findings. She was completely asymptomatic, but transabdominal ultrasound revealed chronic cholecystitis with extremely dilated (>20 mm) common bile duct (CBD) filled with gallstones. Electrocardiogram obtained 2 days before the procedure was normal. Next day she was scheduled for ERCP. In conscious sedation with pethidine (1 mg/ kg i.v.) and midazolam (5 mg i.v.), and premedication with 20 mg i.v. of hyoscine butylbromide (Buscopan) in left lateral position we performed ERCP.

**FIGURE 1:**
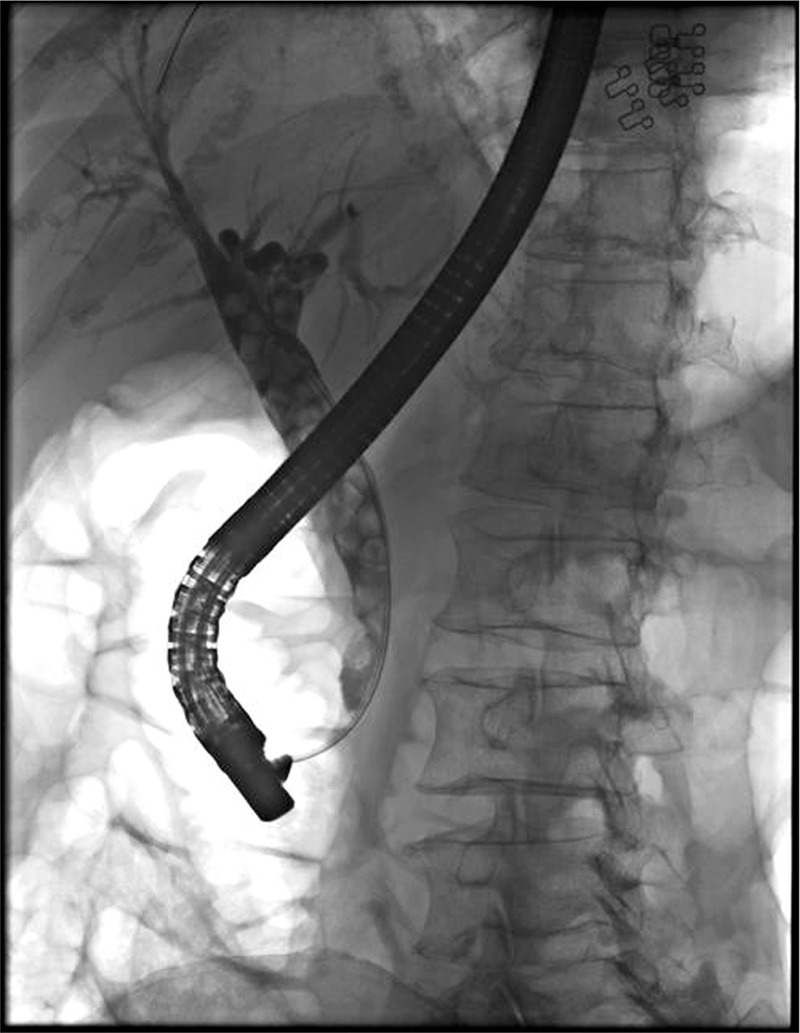
Multiple choledocholithiasis.

After easy cannulation with sphincterotome and guide wire (Cook Fusion Omni-Tome with FSW-35 guide wire) we injected a bolus (10 ml) of iohexol (Omnipaque) contrast solution. X-ray confirmed multiple choledocholitihiasis with extremely dilated CBD (Figure 1). We proceeded with sphincterotomy, which caused spontaneous propulsion of stones. After that we extracted residual stones with a balloon extractor (Cook Fusion Extraction balloon), all together more than 30. During the whole procedure patient was monitored and we recorded no disturbances in cardiac rhythm, blood pressure or oxygen saturation. After we removed the scope patient was awake and we explained to her what we have done.

Several minutes after that, while the patient was still connected to the monitor, we registered alarm due to ventricular fibrillation. Immediately we proceeded with cardiopulmonary reanimation (CPR) according to recent guidelines for Advanced Life Support.^4^ It includes defibrillations, orotracheal intubation and chest compressions. After the third defibrillation, rhythm changed to pulseless electrical activity. Despite prolonged CPR (50 min), the patient died. Autopsy was performed on the next day and it found no obvious cause of sudden death. There were no signs of duodenal wall perforation, myocardial lesions or pulmonary embolism. We suggest that a possible reason of cardiorespiratory arrest could be air embolism of the heart, due to the fact that during autopsy the heart was dissected in a traditional fashion. Any residual air in the heart would then be released into atmosphere upon dissection.

## DISCUSSION

Endoscopic retrograde cholangiopancreatography is a well-established procedure in gastrointestinal medicine used to diagnose and treat many diseases, such as choledocholithiasis or neoplastic lesions of the pancreatobiliary system. Beside the gastrointestinal and pancreatic complications, cardiopulmonary complications of ERCP are also possible and occur in 1% of cases. These include arrhythmias, hypoxemia and transitory myocardial ischemia.^3,5^

Cardiopulmonary and neurological adverse events can occur due to a rare, but potentially fatal complication of ERCP—air embolism. It can lead to hypotension, right heart failure, cardiovascular collapse and even cardiac arrest. Pulmonary manifestations of air embolism can include dyspnea, tachypnea, decrease in end-tidal carbon dioxide (EtCO_2_) concentration, hypoxia, cyanosis, and respiratory failure. Neurological deterioration can be presented as dilated pupil(s), failure to regain consciousness after anesthesia or loss of consciousness, altered mental status, hemiparesis, cerebral edema, etc.^6^

This complication is often neglected and less considered during ERCP. First documented case of air embolism in gastrointestinal endoscopy was described by Lowdon and Tidmore in 1988.^7^ In their report a 5-week-old infant who had biliary atresia due to a previous Kasai procedure died during endoscopy. Autopsy showed presence of air in the right atrium and ventricle and in the area of porta hepatis. However, the infant had a patent foramen ovale and air present in his coronary arteries. Lowdon and Tidmore suggested that air was insufflated across the hepatic tissue into the large hepatic vein, and the patient's heart condition helped development of systemic embolism.

In 2013, Donepudi et al^8^ conducted a systematic review of the risk factors, clinical presentation and management of air embolism in gastrointestinal endoscopy. They found 26 cases of air embolism as a complication of ERCP to date. Almost half of these cases, 12 of them to be precise, ended fatally. Air embolism can occur during any endoscopical intervention in gastroenterology, although most commonly it occurs during ERCP. Air embolism as a complication of ERCP was first noted in 1997 by Kennedy et al.^9^ In their case report, a 63-year-old woman underwent ERCP due to choledocholithiasis. She developed cardiorespiratory arrest due to hepatic venous air embolism. Another case of air embolism during ERCP was described by Mohammedi et al.^10^ A 27-year-old man with a post-traumatic hepatic injury had to have ERCP to determine the location of the leakage of bile. While sphincterotomy was done, a massive air embolism occurred. Luckily, the patient survived. They suggested that insufflated air caused air embolism via damaged hepatic veins.

General consensus about how air embolism occurs is that the air which is insufflated passes intramurally into the portal venous system via duodenal vein radicles which are cut transversely during the procedure. Presence of a biliary-venous fistula or shunt eases the occurrence of air embolism. Other underlying conditions such as previous procedures of the bile duct system, trauma to the liver, gastrointestinal fistulas and inflammations can facilitate air embolism development.^8,10^

Siddiqui et al^11^ reported a case of a fatal air embolism in a 43-year-old female patient after percutaneous liver biopsy and ERCP.

A hypothesis in another air embolism case was made by Stabile et al.^12^ They described a fatal massive paradoxical cerebral embolism in a patient who didn’t have a patent foramen ovale or other septal defects. They proposed that in case of massive air embolism presence, pulmonary circulation could not be capable of filtering all the venous gas, so it could build up on the arterial side and cause ischemia of a certain organ.

Katzgraber et al^13^ reported a case of air embolism in a 56-year-old man who had a gastroscopy because of gastric ulcer. During the procedure he went into cardiac arrest and died. During autopsy air was found in his right ventricle, and also an open vessel at the base of the ulcer. It is notable that air was insufflated at high volumes during the procedure being the possible and likely cause of air embolism.

Finsterer et al^14^ conducted a Medline search in order to provide an overview of current knowledge of pathophysiology, diagnosis, management and prognosis of air embolism during ERCP. They found 18 reports about 19 patients concerning their field of interest. In 14 cases air embolism after ERCP occurred; 8 patients suffered cerebral air embolism and 6 of them ending fatally. According to authors, all these cases were presumed to have air entering blood vessels through portal or hepatic veins. They concluded that in case that patient does not wake up after the procedure, air embolism should be taken into consideration and all of therapeutic measures provided.

Several other case reports describe air embolism during endoscopy procedures. Some patients survived due to recognition of symptoms and swift initialization of treatment like in the case report by Goins et al.^15^ They reported a case of a 72-year-old woman undergoing ERCP because of cholangiocarcinoma. During the procedure she developed pulseless electrical activity and went into cardiac arrest due to a massive air embolus in her right heart. Transesophageal echo found 30 ml of air in her right ventricle. Pulmonary artery catheter was inserted and air was aspired saving the patient's life. They suggest that an injury of a small vessel had occurred and allowed air to pass into the veins which then travelled up to the right heart.

Cha et al^16^ described a case of massive fatal embolism during ERCP. A 50-year-old female went into cardiac arrest during the procedure. Air embolism originated either from a biliary-venous fistula forming due to tissue damage caused by insufflation pressure, or from the previously existent choledochoduodenostomy which could allow easier access for the endoscope and therefore let more air to be insufflated. Another similar case was reported by Bisceglia et al.^17^ A 78-year-old man underwent ERCP for recurrent cholangitis due to gallstones and went into cardiac arrest. Autopsy revealed massive pulmonary and cerebral air embolism, and a spontaneous duodenobiliary fistula which was the entering point for the embolus which then penetrated the intrahepatic veins already weakened due to his disease.

Other case reports suggest cerebral complications due to paradoxical air embolism. Rangappa et al^18^ presented a case of a 50-year-old woman undergoing ERCP due to suspected choledocholithiasis, who suffered a fatal cerebral air embolism. After the procedure the patient was unresponsive with her gaze deviating to the right. CT of her head showed air embolism in her right hemisphere with tonsilar herniation and diffused cerebral edema. During autopsy her heart was placed underwater which showed air in the right atrium. Cause of death was brain edema due to paradoxical air embolism caused by injury of blood vessels at the site of sphincterotomy. Nayagam et al^19^ reported a case of a 56-year-old man who died during ERCP due to cerebral ischemia caused by venous and arterial air embolism. Furthermore, Lopez et al^20^ presented a case of a 61-year-old woman who died of pneumocephalus and ischemic infarction of the right hemisphere. She underwent esophagogastroduodenoscopy due to hematemesis caused by esophageal varices. The patient developed hypotension and was unresponsive. Brain death was declared 24 hours post admission. Another patient suffered from systemic air embolism that presented with deterioration of neurological state. Bechi et al^21^ described a case of a complete neurological recovery in a 79-year-old female patient who developed systemic air embolism during endoscopic sphincterotomy and gallstone removal. They provided only conservative treatment: 100% oxygen, head down position, lateral position and fluid resuscitation.

In a report by Mellado et al^22^ a 52-year-old woman with ovarian cancer suffered from a severe deterioration of her consciousness. A CT scan was made with findings of multiple air embolism areas between the anterior and middle right cerebral arteries. Patient died after 48 hours. Argüelles et al^23^ reported a case of patient who died due to a cerebral artery embolism during ERCP as he developed a severe ishemic brain injury. Another case of cerebral air embolism is described by Laan et al.^24^ A patient had his gastric-mediastinal fistula evaluated following subtotal oesophagectomy and gastric tube reconstruction due to oesophageal cancer. Air embolism developed causing acute left sided hemiparesis. Hyperbaric oxygen was administered, and the patient almost fully recovered.

Most recent case of air embolism during ERCP has been published in 2014. Painter et al^25^ described a case report about a 76-year-old female patient who underwent ERCP to remove a biliary stent and have a lithotripsy. They noted acute drop in end-tidal CO_2_ with cardiorespiratory arrest soon after. They immediately started with advanced cardiac life support and a transesophageal echo was done proving pulmonary embolism. Thrombolytic therapy was administered and the patient was discharged with oral warfarin therapy.

All of the summarized cases describe patients undergoing ERCP and dying from fatal air embolism occurring either in the brain or in the heart.

We can see that air embolism is caused either by high air insuflation pressure or by invasive procedures (ie, sphincterotomy), which can damage the integrity of gastrointestinal structures. Additional risk factor is an existent biliary-hepato-venous fistula or an open/injured blood vessel, as they can provide access to the bloodstream. Air embolism is difficult to recognize, and physicians should be aware of it as an ERCP complication. If a patient suddenly becomes hypoxic, bradycardic, hypotensive, or neurogically decompensated we should always take air embolism into consideration. Transesophageal echocardiography is useful in finding gas in the heart.^16^ Management of air embolism should start by positioning the patient in the Trendelenburg position. Furthermore, hyperbaric oxygen is the therapy of choice. It reduces the gas bubbles’ volume and allows them to dissolve more easily. It also decreases cerebral edema due to hyperoxic vasoconstriction and prevents reperfusion injury by preventing leukocyte aggregation.^18^ Also if necessary, central venous catheter can be of great use, as it can aspirate excess of air from the heart.^15^

## CONCLUSIONS

In our case, sudden cardiac death was unexpected and cause of it unknown. According to known similar cases, we can only suggest that the death of our patient was caused by air embolism. Risk factors for air embolism such as previous procedures of the bile duct system, abdominal trauma or digestive system inflamation should always be considered. Some preventive measures can also be used. For example, using CO_2_ as the insufflating agent instead of air lowers the possibility of embolism, due to easier absorption. End-tidal CO_2_ monitoring is also useful and important. It is a simple, cheap and an effective method. Although prevention of air embolism during ERCP is not difficult, constant education and sharing experiences can teach us to quickly recognize the symptoms of air embolism which is important for the treatment to be successful.
